# G protein‐coupled estrogen receptor agonist G‐1 decreases ADAM10 levels and NLRP3‐inflammasome component activation in response to *Staphylococcus aureus* alpha‐hemolysin

**DOI:** 10.1002/mbo3.1423

**Published:** 2024-06-12

**Authors:** Huayu Zheng, Kathleen D. Triplett, Eric R. Prossnitz, Pamela R. Hall, Seth M. Daly

**Affiliations:** ^1^ Department of Pharmaceutical Sciences University of New Mexico Health Sciences Center, College of Pharmacy Albuquerque New Mexico USA; ^2^ Department of Internal Medicine, School of Medicine, Center of Biomedical Research Excellence in Autophagy, Inflammation and Metabolism and University of New Mexico Comprehensive Cancer Center University of New Mexico Health Sciences Center Albuquerque New Mexico USA

**Keywords:** ADAM10, alpha‐hemolysin, GPER, inflammasome, NLRP3, *Staphylococcus aureus*

## Abstract

The G protein‐coupled estrogen receptor, also known as GPER1 or originally GPR30, is found in various tissues, indicating its diverse functions. It is typically present in immune cells, suggesting its role in regulating immune responses to infectious diseases. Our previous studies have shown that G‐1, a selective GPER agonist, can limit the pathogenesis mediated by *Staphylococcus aureus* alpha‐hemolysin (Hla). It aids in clearing bacteria in a mouse skin infection model and restricts the surface display of the Hla receptor, ADAM10 (a disintegrin and metalloprotease 10) in HaCaT keratinocytes. In this report, we delve into the modulation of GPER in human immune cells in relation to the NLRP3 inflammasome. We used macrophage‐like differentiated THP‐1 cells for our study. We found that treating these cells with G‐1 reduces ATP release, decreases the activity of the caspase‐1 enzyme, and lessens cell death following Hla intoxication. This is likely due to the reduced levels of ADAM10 and NLRP3 proteins, as well as the decreased display of the ADAM10 receptor in the G‐1‐treated THP‐1 cells. Our studies, along with our previous work, suggest the potential therapeutic use of G‐1 in reducing Hla susceptibility in humans. This highlights the importance of GPER in immune regulation and its potential as a therapeutic target.

## Graphical abstract

G‐1, a selective agonist of the G protein‐coupled estrogen receptor (GPER), mitigates the pathogenesis mediated by alpha‐hemolysin, a toxin produced by *Staphylococcus aureus*. It achieves this by reducing the surface display of the toxin's receptor, ADAM10. Our study, conducted on macrophage‐like differentiated THP‐1 cells, reveals that treatment with G‐1 leads to a decrease in ATP release, a reduction in the activity of the caspase‐1 enzyme, and a decrease in cell death following exposure to alpha‐hemolysin.

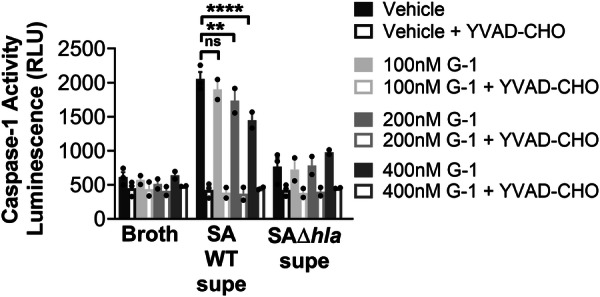

## 1 INTRODUCTION

*Staphylococcus aureus* (SA) is the most common etiology of skin and soft‐tissue infections (SSTIs), that has become increasingly antibiotic‐resistant, that is, methicillin‐resistant SA (MRSA) and vancomycin‐resistant SA (VRSA). SA causes significant morbidity and mortality in the United States and globally, and is listed as an ESKAPE pathogen (*Enterococcus faecium, Staphylococcus aureus, Klebsiella pneumoniae, Acinetobacter baumannii, Pseudomonas aeruginosa*, and Enterobacter spp.). SA utilizes a cornucopia of virulence factors to evade host immune responses, including the toxin alpha‐hemolysin (Hla) which binds ADAM10 and forms membrane pores (Berube & Wardenburg, 2013). We previously demonstrated that G‐1, an agonist of the G protein‐coupled estrogen receptor (GPER) (Arterburn & Prossnitz, 2023; Bologa et al., 2006; Prossnitz & Barton, 2023), limited alpha‐hemolysin (Hla)‐mediated SSTI caused by MRSA in wild‐type (WT) but not GPER knock‐out (GPERKO) mice (Triplett et al., 2019). Improved pathological outcomes during SSTI were associated with significantly reduced interleukin‐1β (IL‐1β) levels in G‐1‐treated mice. This suggested reduced activation of NLRP3 inflammasome. We also reported that G‐1 decreased surface expression of the Hla‐receptor ADAM10 (Wilke & Wardenburg, 2010), on a human keratinocyte cell line.

Similar to other human immune cells, human THP‐1 cells express all components of the NLRP3‐inflammasome (Wang et al., 2020). The NLRP3‐inflammasome is formed by the assembly of NLRP3 oligomers via pyrin domain (PYD) PYD‐PYD interactions with ASC (apoptosis‐associated speck‐like protein containing a CARD) followed by caspase activation and recruitment domain (CARD) CARD‐CARD interactions of ASC and pro‐caspase‐1, leading active of caspase‐1 (Liao et al., 2022). Activated caspase‐1 can then process IL‐1β and IL‐18 for secretion. Activation of the NLRP3‐inflammasome requires both priming and activation steps. Priming can be achieved through pattern recognition receptors, such as TLRs, binding bacterial components, leading to NF‐κB signaling and subsequent transcription of pro‐IL‐1β and NLRP3‐inflammasome components. The secondary, or activation step, can be mediated by a variety of agents, including bacterial pore‐forming toxins (PFTs) and self‐derived extracellular adenosine 5'‐triphosphate (ATP) (Elliott & Sutterwala, 2015). It has been demonstrated that Hla induces NLRP3‐inflammasome activation in human and mouse monocytic cells (Craven et al., 2009). Here we focus on Hla, which is one of the most prominent host‐injurious toxins secreted by SA, though other *S. aureus* virulence factors can also trigger inflammasome activation such as LukAB and *Panton‐Valentine* leucocidin (PVL*)* (Holzinger et al., 2012; Melehani et al., 2015). Furthermore, previous studies have demonstrated that ADAM10, the host receptor for Hla, is required for SA Hla‐mediated activation of the NLRP3 inflammasome in human monocytes, and within that, cell surface expression of ADAM10 plays a key role (Ezekwe et al., 2016).

GPER, also known as GPER1 or originally GPR30 (Arterburn & Prossnitz, 2023; Prossnitz & Barton, 2023), is expressed in an assortment of immune cells, including in vitro‐differentiated and tissue‐resident macrophages (Blasko et al., 2009; Kanda & Watanabe, 2003; Notas et al., 2020). As previously shown, GPER can regulate NLRP3‐inflammasome expression and activation, with GPER knockout mice exhibiting increased NLRP3 expression and activity (Wang et al., 2019). In contrast, G‐1 reduces expression of NLRP3‐inflammasome components, pro‐IL‐1β cleavage, and signaling via NF‐κB, thus reducing pro‐inflammatory cytokine production (Bai et al., 2020; Blasko et al., 2009; Groban et al., 2020). Whether G‐1 reduces NLRP3‐inflammasome activation in human immune cells in response to Hla remains unknown.

Sterile‐filtered wildtype (WT) SA supernatants contain teichoic acids and a myriad of virulence factors, including Hla, that can both prime and activate the NLRP3‐ inflammasome (Craven et al., 2009). Secreted Hla monomers bind ADAM10 on the host cell surface, and undergo a conformational change leading to recruiting more monomers to assemble a heptameric pore in the membrane (Berube & Wardenburg, 2013). This pore is 1–3 nm in size and allows the escape of Ca^2+^, K^+^, and ATP, as well as other low molecular weight molecules, across the host cell membrane (Berube & Wardenburg, 2013). However, whether and how GPER activation with G‐1 impacts the response of human macrophages (Mϕs) to Hla is unknown. To address these questions, we utilized macrophage‐like differentiated THP‐1 cells in the presence of G‐1 or vehicle with or without Hla.

## 2 MATERIALS AND METHODS

### 2.1 *S. aureus* strains

Strains AH1263 and AH1292 (SA WT and SAΔ*agr*, respectively) were generously donated by Dr. Alexander Horswill from the University of Colorado, Anschutz Medical Campus, Denver, CO. The SAΔ*hla* strain, JLB24, was kindly provided by Dr. Jeffery L. Bose from the University of Kansas Medical Center. AH1263 is an erythromycin‐sensitive, plasmid‐cured version of the well‐known USA300 LAC, and AH1292 is the SAΔ*agr* mutant derivative (Boles et al., 2010). LAC is a clinical MRSA strain isolated in Los Angeles County (hence LAC). JLB24 is a further derivative of the AH1263, that is, AH1263 hla::ΦΝΕ with a transposon knocking out the alpha‐hemolysin gene (Bose et al., 2014).

### 2.2 *S. aureus* supernatants

SA WT (containing Hla), SAΔ*hla*, or SAΔ*agr* was grown for 18 h in 50 mL trypticase soy broth (TSB; BD and Co.) in a 250 mL Erlenmeyer flask. Samples were centrifuged at 3000*g* twice before being sterile filtered using a 0.2 µM PES filter (Thermo Scientific). Supernatants were OD600 matched, aliquoted, and frozen at −20°C until use.

### 2.3 Reagents, cell culture and differentiation

Synthesis of G‐1 was performed as previously described (Bologa et al., 2006). G‐1 solubilized in ethanol was stored at −20°C until use. THP‐1 (ATCC, Manassas, VA) and THP‐1 NLRP3 KO cells (Invivogen) were cultured in RPMI (Corning) lacking phenol red and containing 25 mM d‐Glucose, 0.05 mM 2‐mercaptoethanol, 10 mM HEPES, 1 mM sodium pyruvate and 10% (v/v) fetal bovine serum (FBS). Normocin, 100 µg/mL, was added to medium for THP‐1 NLRP3 KO cells only for culturing. Twenty four hours before differentiation, cells were transferred into RPMI complete medium containing charcoal‐stripped FBS (which removes sex steroid hormones). For terminal differentiation into macrophage‐like cells, THP‐1 monocytes were resuspended into RPMI complete medium containing 10% (v/v) charcoal‐stripped FBS, 20 nM phorbol 12‐myristate‐13‐acetate (PMA) (Park et al., 2007), and either G‐1 (at the indicated concentrations) or vehicle control (ethanol). Cells were treated with PMA for 48 h before transferring to new medium lacking PMA for a 24 h resting period. PMA increased cellular adherence and induced a macrophage‐like morphology of the THP‐1 cells as previously described (Park et al., 2007). Both the THP‐1 and the NLRP3 KO cells were used through passages 20–25.

### 2.4 Caspase‐1 assay

The Caspase‐Glo® 1 (Promega) bioluminescent kit was employed to determine active caspase‐1 levels in THP‐1 macrophages. The caspase‐1 specific inhibitor, YVAD‐CHO, was utilized to determine caspase‐1‐specific NLRP3‐inflammasome activation resulting from *S. aureus* supernatant incubation with vehicle or G‐1. For this assay, cells were resuspended at 4 × 10^5^ cells/mL, differentiated with 20 nM PMA and treated with vehicle or G‐1. Cells were seeded in 384‐well white tissue culture plates (Greiner Bio‐one) and incubated at 37°C with 5% CO_2_. After PMA differentiation, the medium was removed and the cells were treated with 25 µL of 1% *S. aureus* supernatants or TSB or 25 µM Nigericin, each containing vehicle or the indicated concentrations of G‐1. Nigericin, a toxin derived from Streptomyces that creates a pore, serves as a positive control, facilitating ion leakage and thereby NLRP3 activation. Treated cells were incubated for 2 h before adding 25 µL of the kit substrate or the substrate + YVAD‐CHO. A SpectraMax i3x (Molecular Devices) taking measurements every 30 min was used for luminescence detection. Analysis was carried out at time points 90‐ or 120‐min post‐substrate addition when the Caspase‐Glo® 1 signal stabilized.

### 2.5 Flow cytometry

Flow cytometry was conducted on THP‐1 macrophages treated with vehicle or G‐1. Specifically, cells were resuspended at 4 × 10^5^ cells/mL in RPMI + 10% charcoal‐stripped FBS and differentiated with 20 nM PMA containing vehicle/G‐1. After incubation for 48 h cells were removed with prewarmed TrypLE Express (Gibco). After washing, cells were resuspended in ice‐cold flow buffer containing PBS, 2% charcoal‐stripped FBS, 2.5 mM EDTA, and 0.1% sodium azide and blocked with Fc Receptor Binding Inhibitor Antibody (Invitrogen) before proceeding with antibody (Ab) staining. PE‐labeled ADAM10 Ab (Biolegend) was used to detect ADAM10 surface levels using a C6 Plus Flow Cytometer (Accuri).

### 2.6 LDH release assay

Cells were seeded at 5 × 10^5^ cells in 200 µL in a 96‐well plate in medium plus vehicle or G‐1 with 20 nM PMA for 48 h, followed by 24 h without PMA. THP‐1 cells were switched into RPMI + 10% charcoal‐stripped FBS and exposed to 2%v/v WT SA or SAΔ*hla* supernatant for 4 h. The culture medium containing any released LDH was collected by centrifugation. Cytotoxicity was measured as LDH release by CytoTox 96 Nonradioactive Cytotoxicity Assay (Promega) according to the manufacturer's directions. Fifty microliters of the sample was transferred to a new 96‐well plate containing 50 µL of CytoTox 96 Reagent and incubated for 30 min at RT. Fifty microliters of Stop Solution was added, and mixed, and the absorbance at 490 nm was measured with a SpectraMax i3x. Cytotoxicity was calculated for WT supernatant minus SAΔ*hla* supernatant and percent cytotoxicity was determined relative to the maximum LDH release control.

### 2.7 ATP release assay

THP‐1 cells were seeded in 200 µL at 10^5^ cells in a 96‐well plate in medium + vehicle or G‐1 at the indicated concentrations and differentiated as described above. Before stimulation by *S. aureus* supernatants, the cells were first rinsed and preincubated in RPMI medium + vehicle/G‐1 without FBS for 1 h to remove cell debris and serum components, since serum components decrease ATP detection (Seminario‐Vida et al., 2009). The cells were then stimulated with 2% TSB, *S. aureus* WT, SAΔhla, or SAΔagr supernatants with vehicle/G‐1. At the indicated time points the supernatants were collected and heated at 98°C for 2 min to inactivate potential nucleotidase activities (Seminario‐Vida et al., 2009). The extracellular ATP concentration was quantified with an ATP Determination Kit (A22066; Molecular Probes), according to the supplied instructions. Ten microliters of supernatant from each sample was mixed with 90 µL of the luciferin‐luciferase reagent, incubated at 28°C for 15 min protected from light, and luminescence was measured using the SpectraMax i3x. The ATP concentration in each sample was determined based on an ATP standard curve.

### 2.8 qRT‐PCR

THP‐1 or THP‐1 NLRP3 KO cells were seeded in 2 mL with 10^6^ cells in a 12‐well plate, differentiated with PMA and treated with vehicle/G‐1. Cells were then stimulated with 1 mL of 1%v/v TSB, SA WT, or SAΔhla supernatants with vehicle/G‐1 for 3 h. Following treatment, supernatants were removed and washed twice with PBS, and RNA was isolated using an RNeasy Mini Kit (Qiagen). Using 0.75 ng of input RNA, cDNA was generated using a High‐Capacity cDNA Reverse Transcription kit (Applied Biosystems) and then diluted 1:20 in Tris‐EDTA Buffer. Quantitative RT‐PCR was performed on a CFX384 Real‐Time System (Biorad) using predesigned SYBR primers (Integrated DNA Technologies). Beta‐actin (actb), beta‐microglobulin (b2m), and hypoxanthine guanine phosphoribosyltransferase (hprt) were all used as endogenous controls. Three experimental replicates were used for analysis and all data were normalized to 1% TSB + vehicle.

### 2.9 Western blot analysis

THP‐1 cells were prepared as in the qRT‐PCR assay above. Cells were washed twice with cold PBS, and then 100 µL of cold RIPA buffer (Alfa Aesar) containing protease inhibitor (ThermoFisher) was added to wells for lysis. Five microliters of specific ADAM10 inhibitor, GI254023X (MilliporeSigma), was used in the RIPA lysis buffer for the ADAM10 WB to avoid degradation of ADAM10 after cell membrane disruption (Brummer et al., 2018). Lysates were harvested by manual dissociation with a cell lifter after 20 min incubation on ice. The supernatants were collected after centrifuging 14,000*g*, 15 min, 4°C and the protein concentration of the lysates was measured using a BCA Protein Assay (ThermoFisher). A total of 10 µg lysate from each sample was electrophoresed on a 4%–12% Bis‐Tris Plus gel in MES buffer (Invitrogen; ThermoFisher) and transferred to a 0.2 µm PVDF membrane (Bio‐Rad). 5% nonfat milk in TBST (150 mM NaCl, 20 mM Tris, pH 7.6, and 0.1% Tween 20) was used as a blocking agent for 90 min at RT. NLRP3 was detected using rabbit antihuman NLRP3 Ab (Abcam) at 1:500; Pro‐caspase‐1 was detected using rabbit antihuman pro‐caspase‐1 Ab (Abcam) at 1:1000; ADAM10 was detected using rabbit antihuman ADAM10 Ab (Abcam) at 1:1000; β‐actin was detected using mouse antihuman/mouse β‐actin Ab (Santa Cruz Biotechnology) at 1:2000. Primary antibodies bound at 4°C overnight. NLRP3, pro‐caspase‐1, and ADAM10 primary Abs were detected with goat antirabbit poly‐HRP Ab (Invitrogen), and β‐actin primary Ab was detected with goat antimouse poly‐HRP antibody (Invitrogen), all at 1:10,000 at RT for 1 h. Each targeted protein was detected separately. Membranes were imaged using SuperSignal West Femto or Pico PLUS Chemiluminescent Substrate (ThermoFisher) on a Protein Simple FluorChem R (ProteinSimple) or ChemiDoc MP Imaging System (Biorad). Band intensity was quantified using Image Studio Lite (v5.2; LI‐COR) or Image Lab software, respectively, and normalized to β‐actin, with the vehicle set to 100%.

### 2.10 Statistical analyses

All statistical evaluations were performed using GraphPad Prism version 9.3.1 for Windows (GraphPad Software). Two groups were analyzed using an Unpaired Students *t* test. Nested ANOVA was used where biological replicates were included with experimental replicates. Comparisons of greater than two groups utilized a one‐way ANOVA with post hoc multiple comparison analyses as described in the figure legends. Comparisons of greater than two groups with more than one variable utilized a two‐way ANOVA with post‐hoc multiple comparison analyses as described in the figure legends. A *p* < 0.05 was considered statistically significant.

## 3 RESULTS

### 3.1 G‐1 reduces caspase‐1 activation in response to hla

**Figure 1:**
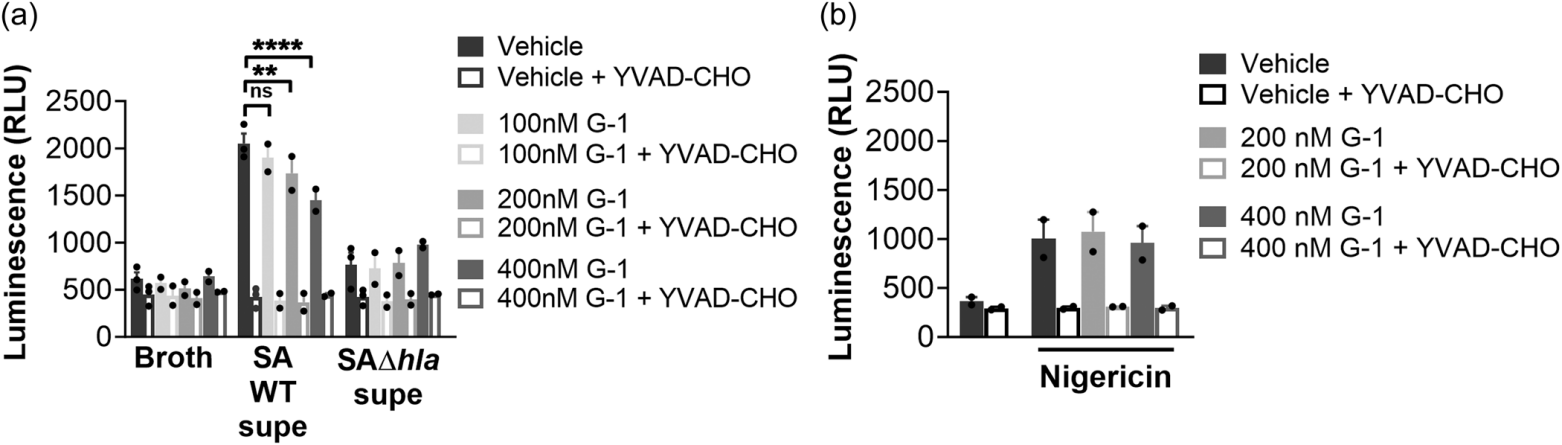
G‐1, concentration‐dependently, reduces caspase‐1 activation by Hla‐containing *Staphylococcus aureus* supernatant. THP‐1 Mϕs, in the presence of Veh or G‐1, were incubated in (a) 1% bacterial medium (broth), SA WT supernatant (Supe) or SAΔhla supernatant, or (b) 20 µM nigericin for 2 h before addition of Caspase‐Glo®‐1 substrate or in the presence or absence of caspase‐1 inhibitor (YVAD‐CHO). Luminescence was measured as described in Section 2. Data shown are mean ± SEM of ≥3 biological replicates from at least two independent experiments. Nested one‐way ANOVA with Šídák's multiple comparison test; **p < 0.01; ****p < 0.0001. ns, not significant.

**Figure A1:**
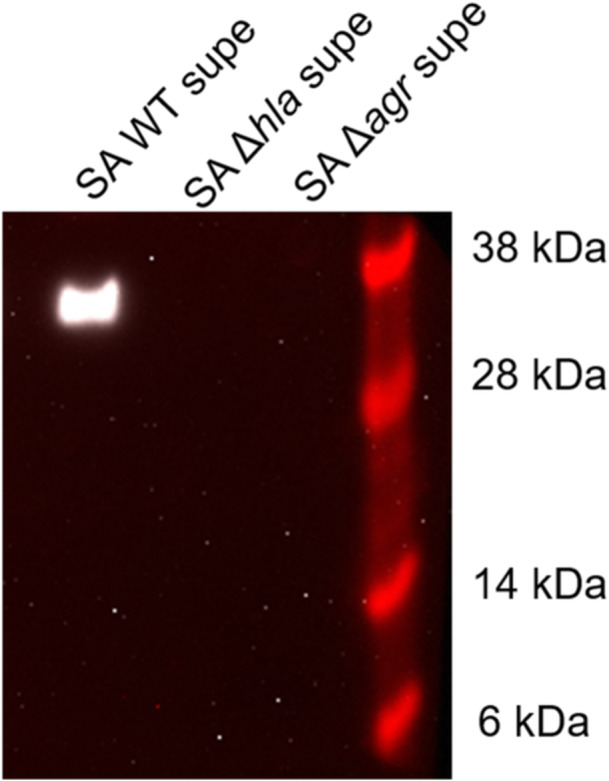
Western Blot analysis of Hla in SA WT, Δ*hla*, and Δ*agr* supernatant. An equal volume of the sterile SA supernatants was electrophoresed on a 4‐12% Bis‐Tris Plus gel in MES buffer (B0002; Invitrogen) before being transferred to 0.2 µm nitrocellulose membrane (Bio‐Rad). Membranes were blocked with 5% nonfat milk in TBST (20 mM Tris, pH 7.6, 150 mM NaCl, and 0.1% Tween 20) for 90 min at RT. Hla was detected using mouse anti‐Staphylococcal alpha‐hemolysin Ab (6C12, IBT Bioservices) at 1:1000 in 5% nonfat milk in TBST at 4°C overnight followed by goat antimouse poly‐HRP Ab (32230, Invitrogen) at 1:10,000 in 5% nonfat milk in TBST at RT for 1 h. Membranes were developed using SuperSignal West Femto Substrate (34095, Thermo Scientific) and imaged on a Protein Simple FluorChem R system (ProteinSimple).

**Figure A2:**
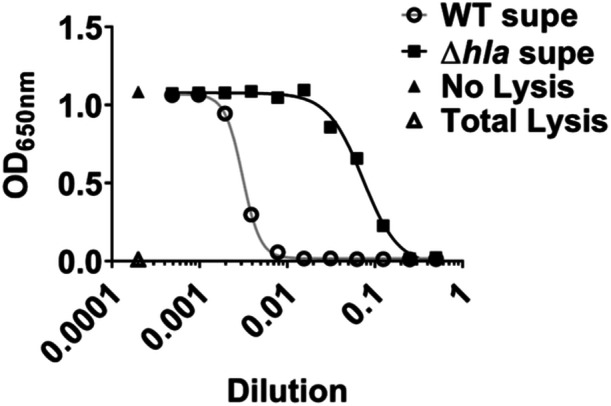
Rabbit red blood cell lysis assay on 18‐h sterile supernatants of SA WT and Δ*hla*. The rabbit red blood cell (RBC) assay was performed as previously described with minor modifications (Bernheimer, 1988). Rabbit RBCs (31081; Colorado Serum Company) were washed twice with cold PBS, diluted to 4% (v/v) in PBS, and 100 µL added to each well of a 96‐well plate. One hundred microliters of SA supernatant was added in the first column and followed by a series of twofold dilutions. One hundred microliters PBS with 100 µL of 4% RBCs, was used as a nonlysis control, and 100 µL PBS + 0.1% Triton with 100 µL of 4% RBCs was used as a total lysis control. The plate was covered with an adhesive seal and incubated at 37°C for 1 h. After 1 h, the plate was shaken to resuspend any RBCs that had not lysed and the optical density was measured at 650 nm in a SpectraMax i3x microplate reader.

To determine if G‐1 alters the sensitivity of human macrophage (Mϕs) to Hla, we utilized THP‐1 cells, differentiated into Mϕs in the presence of PMA. We have previously demonstrated that G‐1 has no direct activity on the growth of SA up to a concentration of 1 µM (Triplett et al., 2019). Using 18 h sterile supernatants from WT SA and a mutant SA lacking Hla (SAΔhla) (Figures A1 and A2), we found that G‐1 concentration‐dependently decreased caspase‐1 activity measured by CaspaseGlo in response to Hla (Figure 1a), while it did not alter the response to either SAΔhla or an unrelated PFT, nigericin (Figure 1b). These results are consistent with our previous results showing reduced IL‐1β at the Hla injection site in G‐1‐treated mice (Triplett et al., 2019). Together, this suggests that G‐1 reduces the activation of caspase‐1 in a concentration‐dependent manner due to the SA‐secreted toxin Hla.

### 3.2 G‐1 reduces ATP release from THP‐1 Mϕs in response to hla

**Figure 2:**
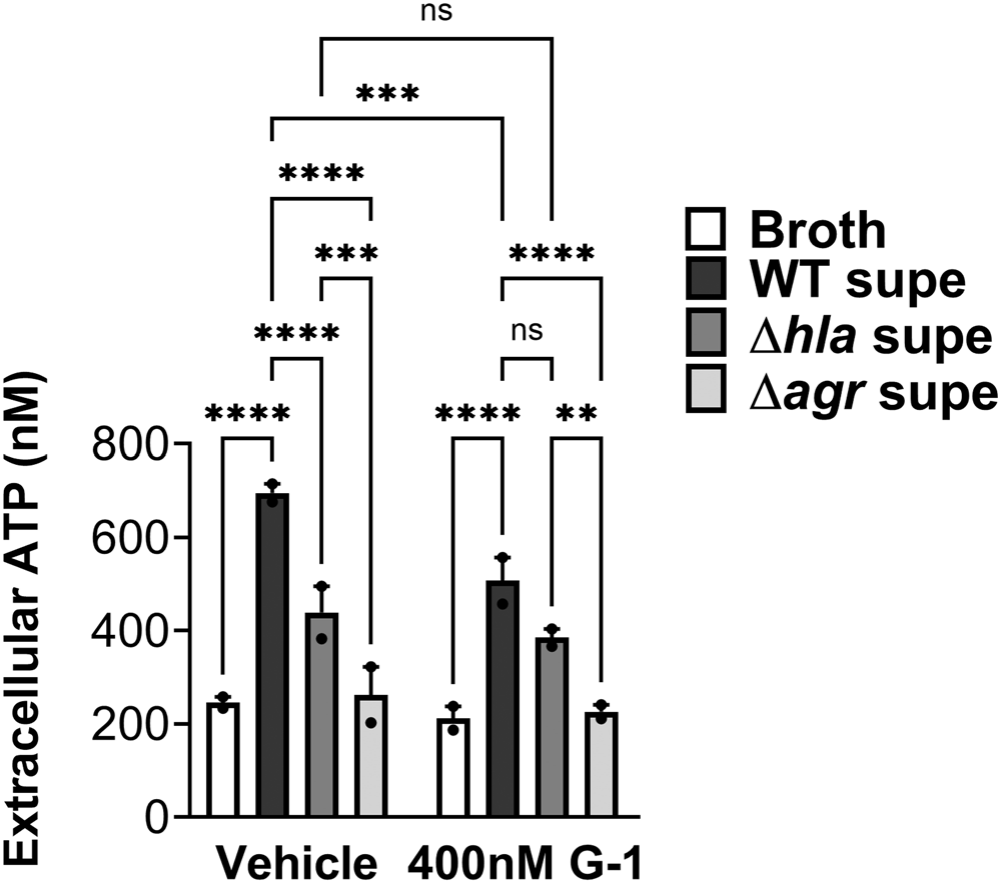
G‐1 reduces Hla‐mediated ATP release from THP‐1 Mϕs. THP‐1 Mϕs were treated with Veh or 400 nM G‐1 and incubated for 2 min with 2% broth or supernatants from WT SA, SAΔhla, or SAΔagr. Data shown are mean ± SEM of ≥3 biological replicates from two independent experiments. Two‐way ANOVA with Tukey's multiple comparison test; **p < 0.01; ***p < 0.001; ****p < 0.0001.

**Figure A3:**
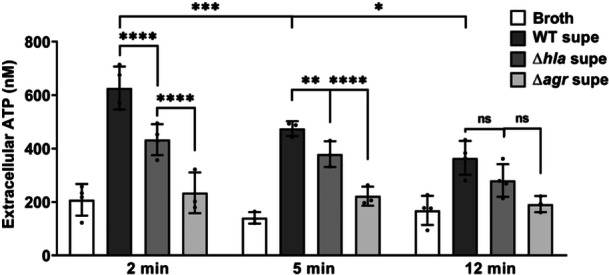
Time‐course for optimal measurement of extracellular ATP release in response to Hla in THP‐1 MΦs. THP‐1 MΦs were incubated with 2% broth or sterile supernatant from SA WT, SAΔ*hla*, or SAΔ*agr* for the indicated times. After incubation, cell culture media was collected, and ATP measured as described in Section 2 and reported as nM ATP based on a standard curve. Data shown are mean ± SEM of *N* = 3–4 technical replicates from a minimum of three independent experiments. Nested One‐way ANOVA with Tukey's multiple comparison test; *p < 0.05; **p < 0.01; ***p < 0.001; ****p < 0.0001. ns, not significant.

Next, we sought to determine if lowered caspase‐1 activation in the presence of G‐1 resulted from reduced ATP release caused by Hla pore formation. Therefore, THP‐1 Mϕs grown with vehicle or 400 nM G‐1, were incubated with 2% broth (control), WT SA supernatant, SAΔhla supernatant, or supernatant from SA lacking expression of Hla and numerous other secreted PFTs (Δagr) (Spaan et al., 2017; Thoendel et al., 2011). To determine the optimal time for measurement, ATP release was first measured extracellularly after 2, 5, and 12 min exposure to broth or WT SA supernatant (Figure A3). Based on comparisons to the standard curve, 2 min was determined to be optimal for measuring extracellular ATP release. At this time point, G‐1 significantly reduced ATP release by THP‐1 Mϕs in response to Hla‐containing WT supernatant (Figure 2). There was a significant difference in ATP release between the SAΔhla and SAΔagr supernatants, due to the additional toxins not expressed in the SAΔagr supernatant (Figure 2). However, G‐1 had no protective effect with either the SAΔhla supernatant or the SAΔagr supernatant (which was not different than broth alone), suggesting the protection is Hla‐dependent. These data support the idea that G‐1 reduces the susceptibility of human Mϕs to Hla toxicity.

### 3.3 G‐1 reduces cytotoxicity of THP‐1 Mϕs in response to hla

**Figure 3:**
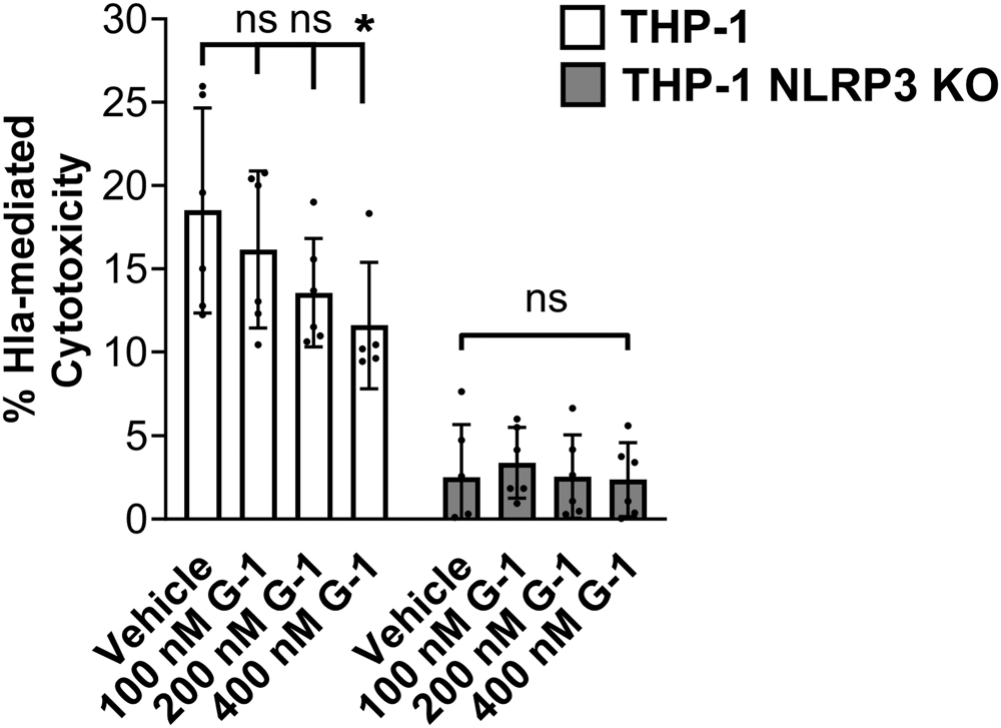
G‐1 prevents Hla‐mediated LDH release via the NLRP3‐dependent inflammasome. THP‐1 NLRP3 KO MΦs treated with vehicle or G‐1 were incubated for 2 h in the presence of 2% WT SA or SAΔ*hla* supernatant before LDH quantitation of the media. Hla‐mediated cytotoxicity was calculated by subtracting LDH release for the Δ*hla* supernatant from that of the WT supernatant, with percent cytotoxicity determined relative to the maximum LDH release control. Data depicted as mean ± SEM of *N* = 3 biological replicates from two independent experiments. Two‐way ANOVA with Dunnett's Multiple Comparison test which compares to the vehicle treatment in each group; *p < 0.05. ns, not significant.

Activation of the NLRP3‐inflammasome can result in pyroptosis, which occurs via cleavage of gasdermin D by caspase‐1 (Yang et al., 2018). We predicted that reduced ATP release and caspase‐1 activation by Hla would result in reduced pyroptosis of THP‐1 Mϕs exposed to Hla. To address this, we measured lactate dehydrogenase (LDH) release following incubation of vehicle‐ or G‐1‐treated THP‐1 Mϕs with supernatant from WT SA. G‐1 treatment reduced LDH release at the 400 nM concentration (Figure 3). This was dependent on NLRP3, as no change was detected in THP‐1 cells lacking NLRP3 (NRLP3 KO). Therefore, GPER activation with G‐1 lowers Hla‐mediated LDH release in an NLRP3‐dependent manner.

### 3.4 Protein, but not mRNA levels, of some NLRP3‐inflammasome components in THP‐1 Mϕs are reduced by G‐1

**Figure 4:**
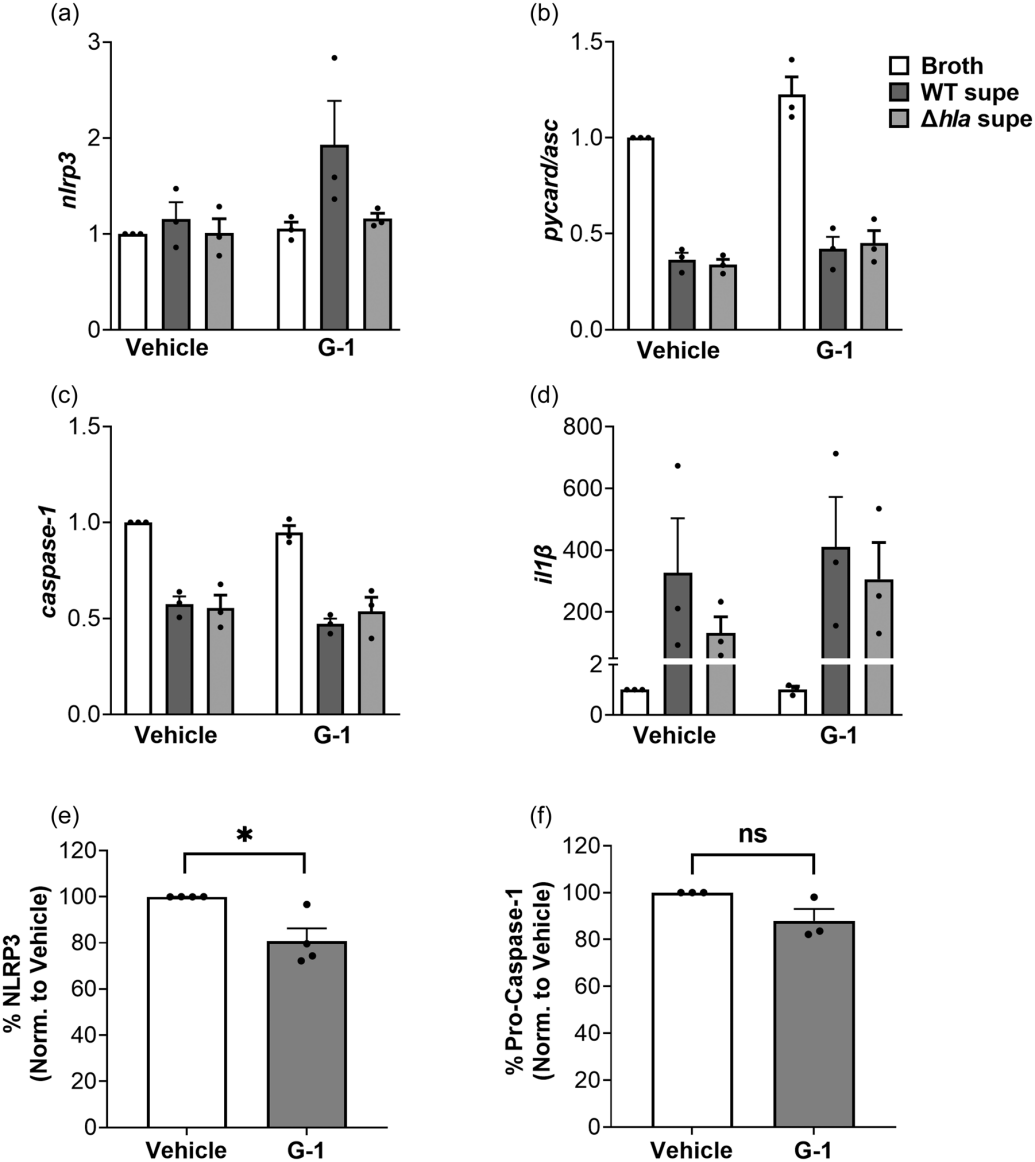
Protein, but not mRNA, levels of some NLRP3‐inflammasome components are altered by G‐1 in THP‐1 MΦs. (a–d) Cells were exposed to Broth control, WT SA supernatant or SAΔ*hla* supernatant for 3 h before RNA isolation and the level of RNA expression was determined by qRT‐PCR. Shown are levels of (a) *nlrp3*, (b) *pycard/asc*, (c) *casp‐1*, and (d) *il‐1β* transcripts. Endogenous controls were actb, b2m, and hprt and data was normalized to broth + vehicle to show fold changes. Data shown are mean ± SEM of *N* = 3 experiments. One‐way ANOVA with Šídák's multiple comparison test; Quantification of Western blots of (e) NLRP3 and (f) Pro‐caspase‐1 levels in THP‐1 MΦs treated with vehicle or 400 nM G‐1 and WT supernatant. Band intensity was compared to β‐actin, with vehicle normalized to 100%. Data shown are mean ± SEM of *N* = 3–4 biological replicates each with 2–4 technical replicates. Nested *t* test; *p < 0.05. ns, not significant.

**Figure A4:**
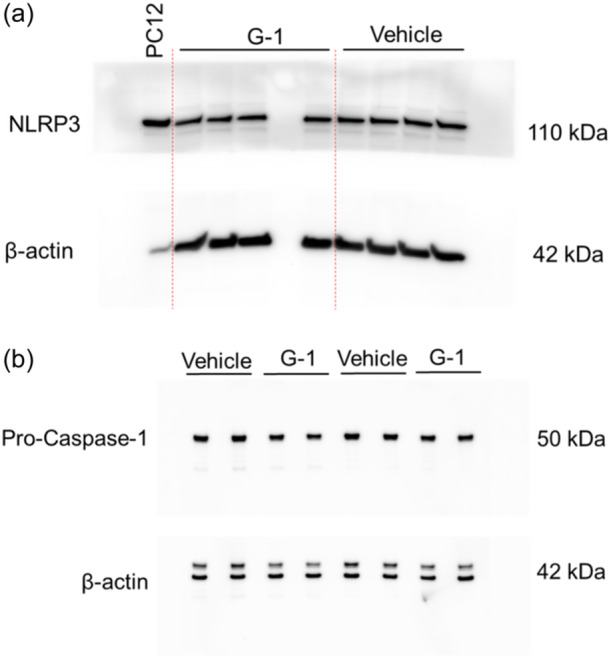
Representative western blot analysis of NLRP3‐inflammasome component levels. The PC12 cell lysate was used as a positive control. (a) NLRP3 and β‐actin were detected separately in the whole cell lysate of vehicle‐ and G‐1‐treated THP‐1 MΦs. (b) The signal was developed for pro‐caspase‐1 first. After that, the membrane was rinsed with TBST and incubated with the secondary antibody for β‐actin followed by the signal development. In the β‐actin image, the bottom bands were the target bands for β‐actin and were used for quantification, and the top bands were for the first developed pro‐caspase‐1. β‐actin was used as a normalized loading control.

**Figure A5:**
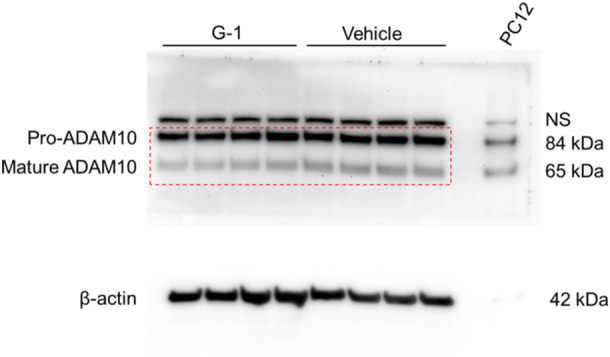
Representative western blot analysis of protein expression of ADAM10. The PC12 cell lysate was used as a positive control. ADAM10 and β‐actin were detected separately. The top bands in the blue box were the off‐targets suggested by the manufacturer's information. Quantifications for mature and pro‐ADAM10 were as described in the Section 2.

Given that G‐1 reduced caspase‐1 activation, ATP release and cell death in THP‐1 MΦs, we asked if G‐1 treatment alone could reduce levels of NLRP3‐inflammasome components. To test this, we first measured mRNA levels, and as shown in Figure 4a–d, mRNA levels of nlrp3, asc, caspase 1 and il‐1β were not altered in the presence of G‐1 compared to vehicle‐treated controls. The only significant effects observed were broth compared to WT supernatant, which was expected but significance was not depicted as we were only interested in the G‐1 effect. We next asked if the total protein of NLRP3‐inflammasome components differed between vehicle‐ and G‐1‐treated THP‐1 MΦs in the absence of Hla. To address this, we measured NLRP3 and pro‐caspase‐1 by Western blot analysis in whole cell lysates of vehicle‐treated and G‐1‐treated THP 1 MΦs. Active caspase‐1 levels were not assessed since the NLRP3 inflammasome was not activated in the absence of Hla. As shown in Figure 4e,f, compared to vehicle‐treated cells, G‐1 reduced NLRP3 protein levels by almost 20% but did not significantly lower levels of pro‐caspase‐1 (Figures A4 and A5). Therefore, consistent with previous findings in the rat hippocampus CA1 region (Bai et al., 2020), G‐1‐ activation of GPER decreases protein levels of the NLRP3 component of the NLRP3‐inflammasome.

### 3.5 G‐1 decreases surface display of ADAM10, the hla receptor

**Figure 5:**
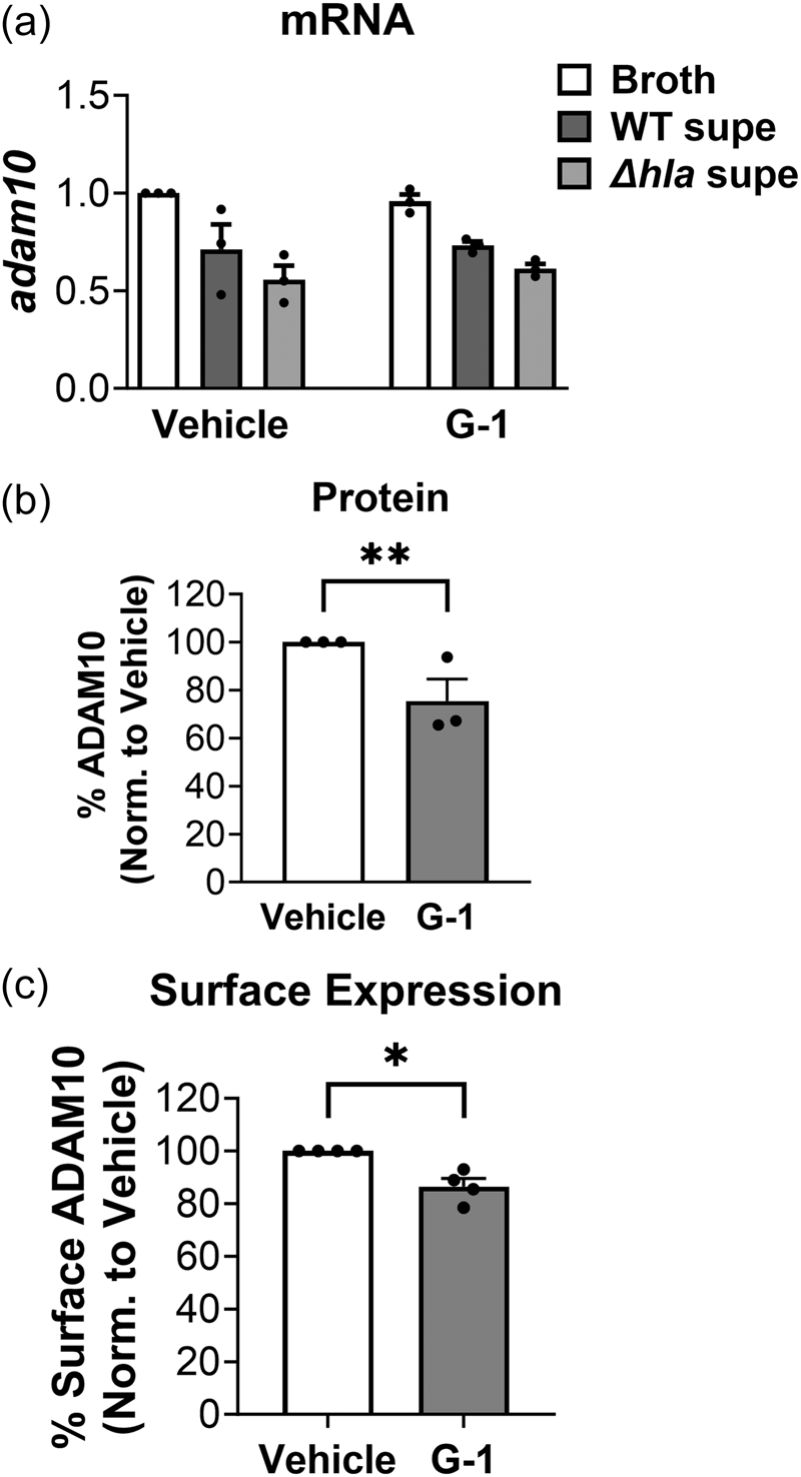
G‐1 reduces ADAM10 surface expression and total protein levels in THP‐1 cells. (a) Cells were dosed with vehicle or 400 nM G‐1 and then exposed to broth control, WT SA supernatant or SAΔ*hla* supernatant for 3 h before qRT‐PCR. Endogenous controls were *actb*, *b2m*, and *hprt* and data were normalized to the broth + vehicle control. Depicted are mean ± SEM of three experiments. One‐way ANOVA with Šídák's multiple comparison test; (b) Quantification of Western blot of ADAM10 levels in THP‐1 MΦs treated with vehicle or 400 nM G‐1. Band intensity was compared to β‐actin as a loading control. Values for vehicle were set to 100%. Data are mean ± SEM of three independent experiments with 2–4 technical replicates. Nested *t* test; **p < 0.01. (c) Surface display by flow cytometry showing significantly decreased surface ADAM10 expression with 400 nM G‐1 treatment. The data shown are the mean ± SEM of four independent experiments. Nested *t* test; *p < 0.05. ns, not significant.

Given our prior observation that G‐1 incubation alone reduced the surface expression of ADAM10 on human keratinocytes (Triplett et al., 2019), we next asked if G‐1 had a similar effect on ADAM10 levels of THP‐1 MΦs. To address this, we measured mRNA, total cellular protein, and cell surface expression of ADAM10 in G‐1‐treated THP‐1 MΦs. Although we did not observe a reduction in ADAM10 mRNA via qRT‐PCR with G‐1 incubation (Figure 5a), we found that overall ADAM10 protein was reduced in these cells through Western blot (Figure 5b), as was cell surface expression assessed by flow cytometry (Figure 5c). Therefore, consistent with our findings using human keratinocytes (Triplett et al., 2019), G‐1 reduced protein levels and surface display of ADAM10 on human immune THP‐1 MΦ cells.

## 4 DISCUSSION

Here we show that G‐1 activation of GPER in human THP‐1 MΦs reduces total protein levels and surface display of the Hla receptor, ADAM10 (Inoshima et al., 2012; Wilke & Wardenburg, 2010), thereby reducing NLRP3‐inflammasome activation. Our previous report demonstrated that G‐1 protected against Hla‐mediated SA skin infection in mice and significantly reduced ADAM10 expression on the surface of a human keratinocyte cell line (Triplett et al., 2019) which is consistent with our current findings. The function of ADAM10 has been attributed primarily to its proteolytic shedding of other surface molecules (Seifert et al., 2020). Although ADAM10 is important for development, inflammation, and cancer, it is also directly involved in response to bacterial infection. ADAM10 is the receptor for Hla produced by SA (Inoshima et al., 2012; Wilke & Wardenburg, 2010), but it is also targeted by PFTs of other bacterial pathogens, such as ExlA of *Pseudomonas aeruginosa* and ShlA of *Serratia marcescens* (Reboud et al., 2017). Furthermore, the type III secreted effector Map from Enteropathogenic *Escherichia coli* (EPEC) triggers the activity of ADAM10, which may contribute to colonization and infection (Ramachandran et al., 2020). Thus, an agent that reduces ADAM10 protein and surface expression levels on multiple cell types may prove beneficial to human health. We also show that G‐1 reduces expression of NLRP3 and caspase‐1 activation in an Hla‐dependent manner, in human THP‐1 MΦs. As previously described, NLRP3‐inflammasome assembly is mediated by the binding of NLRP3, ASC and pro‐caspase‐1, which leads to caspase‐1 cleavage/activation and cleavage/release of IL‐1β. Using hearts from cardiomyocyte‐specific GPER KO mice, Wang et al. showed that loss of GPER increased NLRP3 levels (Wang et al., 2019). Furthermore, in vivo studies demonstrated that cardiomyocyte‐specific GPER KO mice treated with an NLRP3 inhibitor displayed reduced heart disease compared to vehicle‐treated controls (Wang et al., 2019), confirming the role of NLRP3 in heart disease in the absence of GPER. Small molecules that inhibit the NLRP3‐inflammasome represent an ongoing focus of research (Artlett, 2022; Zahid et al., 2019), as the NLRP3‐inflammasome is linked to numerous diseases, such as atherosclerosis, cancer, multiple sclerosis, and ischemia‐reperfusion injury (Wang et al., 2022; Zahid et al., 2019). Direct inhibitors of NLRP3 block its ATPase activity or interaction with binding partners (Zahid et al., 2019). Although in our current study mRNA levels of NLRP3‐inflammasome components did not differ with G‐1 treatment, we did observe a reduction in NLRP3 protein levels in THP‐1 cells due to G‐1 treatment. While the mechanism behind the impact of GPER activation on NLRP3 protein levels requires further investigation, our results suggest a role for GPER in NLRP3 regulation.

We have previously shown that G‐1‐treatment reduced IL‐1β release caused by Hla in a mouse model of SSTI (Triplett et al., 2019). Given that Hla‐mediated pore formation activates the NLRP3‐inflammasome, and downstream activation of caspase‐1, and IL‐1β release, it is reasonable to expect that reduced NLRP3 expression, as seen here, can lead to reduced caspase‐1 assembly and activation, and thus reduced IL‐1β secretion. Similarly, ovariectomized rats treated with G‐1 (Bai et al., 2020) showed reduced expression of components of the NLRP3‐inflammasome after ischemia and reperfusion (IR). These effects were reversed by G36, a highly selective GPER antagonist (Dennis et al., 2011). However, reduced NLRP3 protein expression, demonstrated here in G‐1‐treated immune cells, was not sufficient to inhibit caspase‐1 activation with the potassium ionophore, nigericin, indicating that slightly reduced steady‐state NLRP3 protein levels may not be physiologically relevant. Given that nigericin does not utilize ADAM10 for NLRP3 activation, our findings suggest that the primary mechanism of G‐1 reducing Hla‐mediated caspase‐1 activation in these studies may be through lowering protein levels and surface expression of ADAM10. Also, as certain properties may differ between cultured and immortalized cells, such as the THP‐1 cells used here, and primary human immune cells (Tedesco et al., 2018), testing the effects of G‐1 in reducing ADAM10 expression awaits confirmation in human primary phagocytes. Here, we add our data on the actions of G‐1 on GPER‐mediated defence against Hla in human immune cells. Our findings suggest that G‐1 limits human bacterial toxin‐mediated disease and point to therapeutic applications of the GPER agonist G‐1.

## AUTHOR CONTRIBUTIONS

**Huayu Zheng**: Investigation; writing—original draft; writing—review & editing. **Kathleen D. Triplett**: Investigation; writing—original draft; writing—review & editing. **Eric R. Prossnitz**: Conceptualization; funding acquisition; writing—review & editing. **Pamela R. Hall**: Funding acquisition; conceptualization; writing—original draft; writing—review & editing. **Seth M. Daly**: Investigation; writing—review & editing.

## CONFLICTS OF INTEREST STATEMENT

E. R. P. holds inventorship on two US patents, numbers 7875721 and 8487100. These patents pertain to GPER‐selective ligands and imaging agents, as well as their therapeutic applications targeting GPER. Additionally, both P. R. H. and E. R. P. are credited as inventors on US patent number 10561648. This patent is for the therapeutic use of sex‐specific compounds that target GPER, specifically for the treatment and prevention of bacterial infections.

## ETHICS STATEMENT

None required.

## Data Availability

All data are contained within the article and its Appendix.
